# Evaluating Single‐Spacecraft Observations of Planetary Magnetotails With Simple Monte Carlo Simulations: 1. Spatial Distributions of the Neutral Line

**DOI:** 10.1029/2018JA025958

**Published:** 2018-12-22

**Authors:** A. W. Smith, C. M. Jackman, C. M. Frohmaier, J. C. Coxon, J. A. Slavin, R. C. Fear

**Affiliations:** ^1^ Department of Physics and Astronomy University of Southampton Southampton UK; ^2^ Institute of Cosmology and Gravitation University of Portsmouth Portsmouth UK; ^3^ Climate and Space Sciences and Engineering University of Michigan Ann Arbor MI USA

**Keywords:** flux ropes, Mercury, MESSENGER, Monte Carlo, reconnection, magnetotail

## Abstract

A simple Monte Carlo model is presented that considers the effects of spacecraft orbital sampling on the inferred distribution of magnetic flux ropes, generated through magnetic reconnection in the magnetotail current sheet. When generalized, the model allows the determination of the number of orbits required to constrain the underlying population of structures: It is able to quantify this as a function of the physical parameters of the structures (e.g., azimuthal extent and probability of generation). The model is shown adapted to the Hermean magnetotail, where the outputs are compared to the results of a recent survey. This comparison suggests that the center of Mercury's neutral line is located dawnward of midnight by $0.37_{-1.02}^{+1.21}\;R_{M}$ and that the flux ropes are most likely to be wide azimuthally (∼50% of the width of the Hermean tail). The downtail location of the neutral line is not self‐consistent or in agreement with previous (independent) studies unless dissipation terms are included planetward of the reconnection site; potential physical explanations are discussed. In the future the model could be adapted to other environments, for example, the dayside magnetopause or other planetary magnetotails.

## Key Points

- Monte Carlo model allows the estimation of X‐line location and reconnection frequency given sampling with a single spacecraft
- Mercury's magnetotail reconnection site is consistent with a center offset ( $0.37_{\text{‐}1.02}^{+1.21}R_{M}$) dawnward of midnight
- Mercury's downtail X‐line location is only self‐consistent if dissipation terms are included planetward of the X‐line

## 1 Introduction

Magnetic reconnection is the fundamental physical process by which magnetic fields can be reconfigured and, in so doing, transfer stored magnetic energy to the local plasma. Though the phenomenon occurs on very small spatial scales (e.g., Øieroset et al., 2001), it can result in the generation of large magnetic structures, for example, magnetic flux ropes (Hughes & Sibeck, 1987; Moldwin & Hughes, 1991; Russell & Elphic, 1978). Such large‐scale structures can be used to indirectly track the process. For planets with a strong solar wind influence reconnection is also responsible for a cycle of global convection: On the dayside of a planet magnetospheric flux can be opened through reconnection with the interplanetary magnetic field. The newly opened flux can then convect across the poles of the planet with the motion of the solar wind. Open magnetospheric flux can later be closed through reconnection at the center of the magnetotail, allowing the freshly closed field to convect back around to the dayside, completing the cycle (Dungey, 1961).

Flux ropes are helical magnetic structures that can be generated by reconnection at multiple points within a magnetospheric current layer, for example, on the dayside magnetopause (e.g., Lee & Fu, 1985; Russell & Elphic, 1978; Southwood et al., 1988) or at the center of the magnetotail plasma sheet (e.g., Moldwin & Hughes, 1991; Sibeck et al., 1984; Slavin et al., 1989, 1993, 1995). Once generated by reconnection, the direction of motion of the flux ropes is thought to be determined by their location relative to the dominant X‐line, or neutral line. In the magnetotail, those flux ropes planetward of the dominant neutral line move toward the planet and eventually re‐reconnect with the strong planetward field (Slavin et al., 2003), perhaps forming dipolarization fronts (Lu et al., 2015). Meanwhile, tailward of the neutral line flux ropes are ejected down the magnetotail and are lost to the solar wind (e.g., Hones et al., 1984; Ieda et al., 1998; Moldwin & Hughes, 1992). In general, the velocity of the flux ropes far exceeds the orbital velocity of spacecraft, such that spacecraft can be approximated as stationary during a flux rope encounter.

In situ flux rope encounters possess distinctive magnetic field signatures: a bipolar field signature in the normal component and a peak in the axial component and total field strength. In general, these features can be used to identify in situ spacecraft encounters. However, the exact signature is dependent on the relative trajectory of the spacecraft through the structure: examples of several possible trajectories can be found in Borg et al. (2012) and DiBraccio et al. (2015). In general though, the leading and trailing hemispheres of the flux rope are responsible for the extremes of the bipolar signature; if one hemisphere is “missed,” then the signature may be asymmetric. The magnitude of the bipolar signature and peak in the axial direction will strongly depend on the minimum separation between the spacecraft and the center of the structure.

Many magnetotail surveys have been undertaken, using many years of spacecraft data, to identify flux rope signatures and evaluate their properties and distributions. Such surveys have been performed at Earth (e.g., Imber et al., 2011; Moldwin & Hughes, 1992; Slavin et al., 2003), Mercury (e.g., DiBraccio et al., 2015; Smith et al., 2017; Sun et al., 2016), and Mars (e.g., Briggs et al., 2011; Vignes et al., 2004). However, surveys of in situ spacecraft data are inherently limited by the orbital coverage of the spacecraft and ultimately represent single point observations of a very large, stochastic system. This report describes a Monte Carlo‐based approach designed to assess and quantify the impact of orbital sampling on statistical surveys of flux ropes, allowing an estimation of the underlying (or intrinsic) distribution and recurrence rate. These properties are crucial to determine the links between magnetotail conditions (or solar wind driving) and the process of reconnection. The Monte Carlo technique presented in this study has been developed with reference to Mercury's magnetotail but would be applicable to other planetary environments (e.g., other magnetotails or even perhaps magnetopauses) with some adaptation. The inherent biases that are created by placing selection criteria on the required magnetic field signatures are investigated in a companion paper (Smith, Jackman, Frohmaier, et al., 2018).

### 1.1 Mercury's Magnetotail

Data from the flyby of Mariner 10 suggested that the Near Mercury Neutral Line (NMNL) was located between 3 and 6*R*_*M*_ (*R*_*M*_=2,440 km) down the magnetotail. Later, during two flybys of the National Aeronautics and Space Administration (NASA)'s MESSENGER (MErcury Surface, Space ENvironment, GEochemistry and Ranging) spacecraft (M2 and M3), the neutral line was inferred to be 2.8 and 1.8*R*_*M*_ from the planet, respectively (Slavin et al., 2012), using the orientation of the magnetic signatures of flux ropes. MESSENGER later orbited Mercury between March 2011 and April 2015 (Solomon et al., 2007), collecting high‐resolution magnetometer data (Anderson et al., 2007). MESSENGER's orbit was highly inclined and elliptical with an 8‐ to 12‐hr period (depending on the phase of the mission). The orbit precessed around the planet once every Mercury year (∼88 days), such that the spacecraft made cuts through the magnetotail plasma sheet approximately twice per day during “hot” and “warm” seasons. These plasma sheet crossings generally lasted less than 10 min (Poh et al., 2017a), a period during which flux ropes were often observed to pass over the spacecraft as they moved tailward/sunward from the location at which they were generated (assumed to be in close proximity to the NMNL).

A statistical analysis of magnetometer data from 319 of MESSENGER's plasma sheet crossings has suggested that the NMNL is most often located ∼3*R*_*M*_ down the tail (Poh et al., 2017a). However, complementary studies of large numbers of flux ropes (and their inferred direction of travel) have been less clear, perhaps suggesting a large degree of variability in the downtail location of the NMNL (DiBraccio et al., 2015; Smith et al., 2017). In addition to inferring the approximate location of the NMNL, statistical flux rope surveys (e.g., Smith et al., 2017; Sun et al., 2016) have noted a dawnward offset in the observed flux rope distributions. This also correlates with shifts in statistical field distributions (Poh et al., 2017b), dipolarizations (Dewey et al., 2017), and the distribution of energetic electrons (Baker et al., 2016) and their precipitation onto the surface (Lindsay et al., 2015). In addition, Zhong et al. (2018) recently reported the first observations of an active reconnection site in Mercury's magnetotail, during which the spacecraft was located ∼0.5*R*_*M*_ dawnward of midnight.

Smith et al. (2017) investigated the number of flux ropes observed during plasma sheet crossings, as well as the spacing between consecutive observations. The majority of crossings (61%) did not feature any flux ropes, while groups of up to eight were observed during periods of intense activity. Meanwhile, the spacing between adjacent flux ropes was generally found to be less than 100 s, and therefore, consecutive events could be related to the same interval of reconnection. For context, the Dungey cycle timescale at Mercury is thought to be very short, perhaps as little as 2 min (Christon, 1987; Siscoe et al., 1975; Slavin et al., 2009, 2012). Similarly, the duration of Hermean substorms has been found to be ∼200 s on average (Imber & Slavin, 2017).

Section 2 describes the setup of the Monte Carlo model. Section 3 then considers the general results of the model, investigating the effects of varying the model parameters and the orbital selection. Section 4 then compares the results of the model to those of a recent large survey (Smith et al., 2017), allowing investigation of the intrinsic properties Mercury system (including neutral line location and width).

## 2 The Model

In this section the design and properties of the model will be discussed, along with the some of the implicit assumptions of such a setup.

### 2.1 Model Setup

The orbit of MESSENGER resulted in plasma sheet crossings that were separated by ∼8–12 hr, much longer than the timescale on which global Hermean magnetospheric dynamics operate. Additionally, during just under half of all MESSENGER plasma sheet crossings there were short periods during which the products of a (likely single) reconnection interval could be observed (Smith et al., 2017). Therefore, for the purposes of this work we will treat each plasma sheet crossing as independent (from adjacent crossings) and assume that (at most) one instance of tail reconnection can occur. If this model were adapted for comparison with other surveys/environments, then the validity of these assumptions would need to be reevaluated.

The Cartesian Mercury Solar Magnetospheric (MSM) coordinate system is used in this study. In this system, the ${\hat{X}}_{\text{MSM}}$ axis points toward the Sun, the ${\hat{Z}}_{\text{MSM}}$ axis is aligned with the magnetic dipole and directed northward, and the ${Ŷ}_{\text{MSM}}$ axis completes the right‐handed set (pointing duskward). The model forms a two‐dimensional plane (the equivalent of the *X*_MSM_‐*Y*_MSM_ plane), approximating the plasma sheet on the nightside of the planet. The model is set up to simulate a given number of orbits, which are approximated as vertical passages through the plasma sheet to approximate the trajectory of MESSENGER. Therefore, for each orbit the spacecraft location (*X*_MSM_ and *Y*_MSM_) and plasma sheet dwell time are generated. The location is initially drawn from a uniform distribution, while the dwell time is drawn from a database of current sheet crossings identified in the MESSENGER data (Poh et al., 2017a). This initial setup simulates a spacecraft data set with completely even coverage (i.e., with no orbital bias), which may represent the ideal scenario for a large statistical survey. During a fraction of orbits (an adjustable parameter) reconnection is deemed to have occurred. Initially the probability is set to 50% of orbital passes, and for each of these a neutral line is generated. The effects of changing this probability will be explored further in sections 3 and 4. In reality, the probability of observing a flux rope during a crossing of the Hermean plasma sheet has been found to scale with the magnitude of the preceding lobe magnetic field strength (Smith et al., 2017). The generated neutral lines have a randomly selected center (*X*_MSM_ and *Y*_MSM_) and azimuthal width (*W*_NL_). It should be noted that while the neutral line in the model is implicitly assumed to be stationary during each plasma sheet crossing, neutral lines have commonly been observed to retreat tailward at Earth (e.g., Alexandrova et al., 2015; Eastwood et al., 2010), Jupiter (e.g., Kasahara et al., 2011; Kronberg et al., 2005), and Saturn (e.g., Smith, Jackman, Thomsen, et al., 2018). Limiting the azimuthal width of the neutral line implies the presence and closure of field aligned currents. Such field aligned currents have been observed by MESSENGER and are postulated to close through the conducting interior of the planet (e.g., Anderson et al., 2014). The results of global magnetohydrodynamic modeling are also consistent with such current systems (Jia et al., 2011).

**Figure 1:**
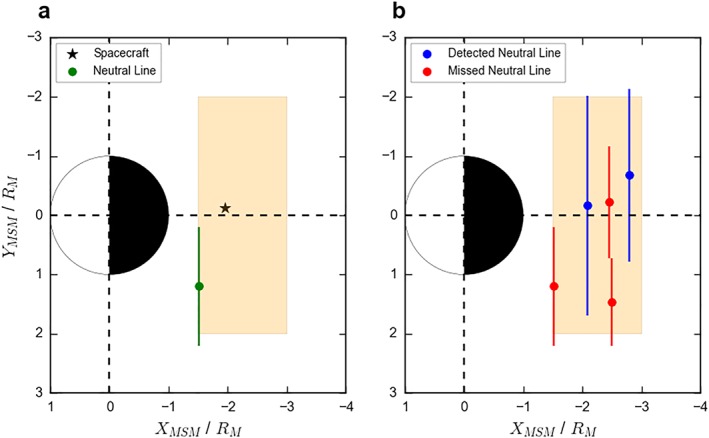
Schematic describing the model setup. Panel (a) shows an example orbit, with a randomly generated spacecraft location (black star) and neutral line (in green). The orange shaded region shows the limit of the uniform distributions used to generate the orbits and neutral line centers. Panel (b) shows the results of 10 orbits where the reconnection probability has been set to 50%. The blue neutral lines show those that were spatially coincident with the generated spacecraft locations during that orbit, while the red neutral lines show those that were missed by their respective spacecraft. MSM = Mercury Solar Magnetospheric coordinate.

This setup is illustrated in Figure 1a. The orange shaded area shows the region within which the spacecraft and neutral line could be generated, roughly representing MESSENGER's coverage of Mercury's magnetotail. An example generated spacecraft location (black star) and neutral line (green point and line) are shown in Figure 1a.

As a first approximation, the neutral line is considered to generate a single flux rope moving planetward and a single flux rope moving tailward, with azimuthal widths provided by the extent of the neutral line. If the neutral line and spacecraft are spatially coincident (along the Y_MSM_ axis) then the neutral line is considered to be “detected.” Selection effects, that is, those that would cause the flux rope to not be identified even when encountering the spacecraft, are considered in a companion paper (Smith, Jackman, Frohmaier, et al., 2018). With this setup the number of flux ropes generated either side of the stationary neutral line is equal, supported by the approximately equal numbers of planetward and tailward moving flux ropes observed by recent surveys (DiBraccio et al., 2015; Smith et al., 2017). Consideration of the impact of neutral line motion and the generation of multiple flux ropes is outside the scope of this paper but could be considered in future adaptations of this model.

The model allows a map to be constructed where flux ropes (and associated neutral lines) are detected and where they are missed, purely as a result of the spacecraft coverage. Figure 1b shows the results of 10 orbits. Five neutral lines have been generated (i.e., 50% of the orbits are associated with reconnection). The red neutral lines show those that were not spatially coincident with their respective spacecraft and so were missed, while the blue neutral lines show those that generated flux ropes that passed over the randomly placed spacecraft. In accordance with expectation, though with a small sample size, it can be seen in Figure 1b that the wider neutral lines were detected, while the smaller ones were missed by the random sampling. This effect will be further explored in sections 3 and 4.

It should be noted that no boundary effects are considered (e.g., the dawn or dusk magnetopause). Instead, the boundaries are implicitly provided by the limits of the spacecraft orbit and neutral line centers simulated. This does mean that some portion of the neutral line width may be outside of the region within which the spacecraft could observe it. Therefore, if the center of the neutral line is placed at the edge of the spacecraft's orbital region then the effective length of the neutral line could be up to a factor of two shorter than that explicitly generated.

## 3 Recovery of the Intrinsic Distribution

To begin, the distributions that are recovered by (or inferred from) the virtual spacecraft will be compared to those that would be obtained with complete magnetotail coverage (i.e., the true or intrinsic distribution). This provides a measure of the effectiveness of the spacecraft sampling and can be evaluated as a function of the number of orbits, orbital selection, or properties of the dynamic structures of interest (e.g., recurrence or extent).

### 3.1 Increasing the Number of Orbits

In this section, the model results will be discussed while drawing the spacecraft position ( $X_{\text{MSM}}^{\text{SC}}$, $Y_{\text{MSM}}^{\text{SC}}$), neutral line center ( $X_{\text{MSM}}^{\text{NMNL}}$, $Y_{\text{MSM}}^{\text{NMNL}}$), and neutral line width (W_NL_) from uniform distributions, the details of which are provided in Table 1. The reconnection probability is initially set to 0.5. It should be noted that these correspond to initial test parameters, selected to demonstrate the effects of increasing the random sampling. The parameters will be further investigated in section 3.2.

**Table 1:** The Distributions From Which Draw Parameters Were Drawn in Section 3

Parameter	Distribution	Minimum	Maximum
$X_{\text{MSM}}^{\text{SC}}$	Uniform	−3R_M_	−1.5R_M_
$Y_{\text{MSM}}^{\text{SC}}$	Uniform	−2R_M_	2R_M_
$X_{\text{MSM}}^{\text{NMNL}}$	Uniform	−3R_M_	−1.5R_M_
$Y_{\text{MSM}}^{\text{NMNL}}$	Uniform	−2R_M_	2R_M_
W_NL_	Uniform	2R_M_	2.5R_M_

**Figure 2:**
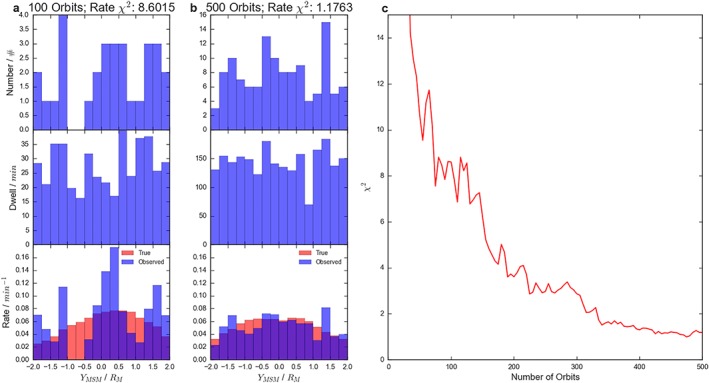
Figure showing how the observed/inferred rate of flux ropes measured across the model magnetotail compare to the “true” distribution after 100 orbits (a), 500 orbits (b), and as a function of orbits (c). For the left and center panels the top row shows the number of flux ropes observed per bin, the middle shows the cumulative spacecraft dwell time, and the bottom shows the inferred rate (blue) and true rate (red) given complete spacecraft coverage. The model parameters are provided in Table 1. MSM = Mercury Solar Magnetospheric coordinate.

As orbits are added it is possible to build dawn‐dusk maps of the distribution of flux ropes observed. Figure 2 explores how the addition of orbits affects the comparison between the inferred and “true” distributions (i.e., the distribution that would be obtained if the entire tail were monitored by spacecraft). Figures 2a and 2b show the results after 100 and 500 randomly distributed orbits, respectively. The top panels show the number of flux ropes observed by the spacecraft, the middle shows the spacecraft cumulative dwell time, while the bottom shows the inferred rate in blue. The red bars in the lower panels represent the distribution that would be inferred if the observations of multiple spacecraft (evenly spaced across the entire magnetotail) were combined, that is, the true distribution. It is possible to compare the recovered and true distributions using a χ ^2^ metric; the values of which are shown above Figures 2a and 2b. The lower the value of this measure, the closer the observed rate matches the value that would be recovered with complete magnetotail coverage. A value approaching 1 would suggest good agreement.

Between 100 and 500 orbits the intrinsic/true distributions (red) do not change significantly: the underlying distribution is fairly settled. However, after 100 orbits have been completed the randomly located spacecraft has not adequately sampled the tail, and so the *χ* ^2^ is high: the observed distribution poorly represents the underlying distribution. In contrast, once 500 orbits have been performed, the system has been much better sampled, and the *χ* ^2^ has dropped by a factor of ∼8.

Figure 2c shows how the *χ* ^2^ (between the true and inferred distributions) varies as a function of the number of orbital passes. Overall, the *χ* ^2^ can be seen to drop rapidly with the addition of more orbits. Eventually, this effect is saturated and the *χ* ^2^ plateaus after ∼300–350 uniformly distributed orbits. There are some exceptions to this behavior, with small jumps observed, perhaps when a region is temporarily over sampled and the stochastic nature of the modeled reconnection boosts the rate in a region to an unrepresentative value.

**Figure 3:**
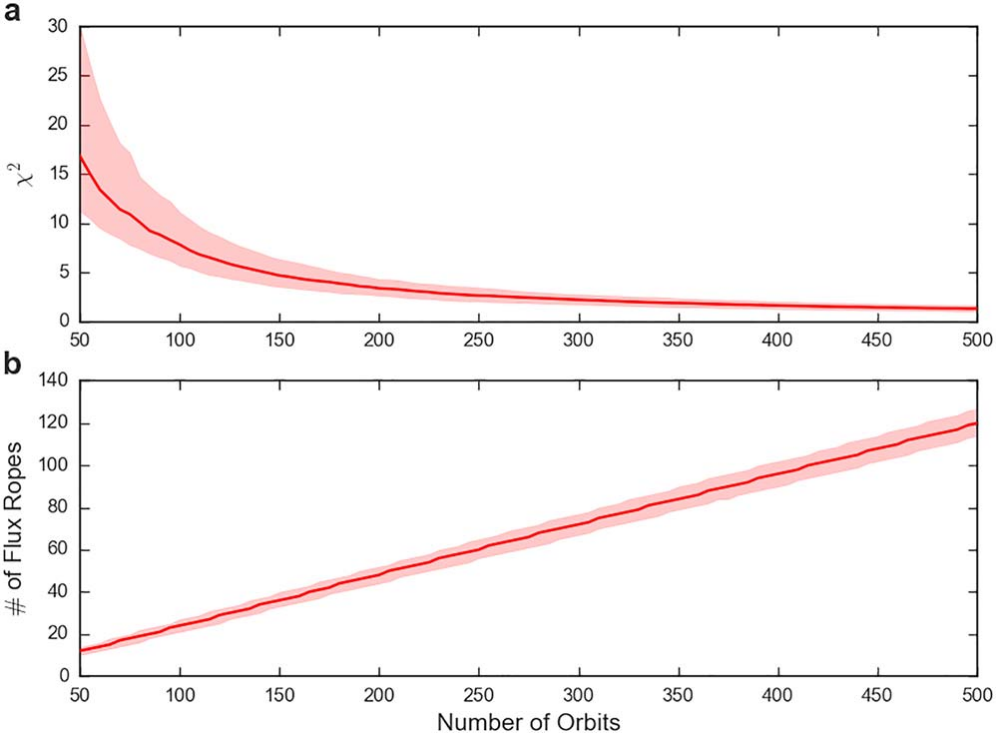
Figure showing the median χ ^2^ between the inferred and “true” cross‐tail distributions (a) and median number of flux ropes observed (b) as a function of the number of orbits performed (after 1,000 iterations of the model). The limits of the red shaded region represent the interquartile range. The model parameters are provided in Table 1.

Figure 3a shows the median variation in *χ* ^2^ as a function of orbits (for 1000 sets of orbital passes, or iterations, which has the effect of removing the random fluctuations). It can be seen that the value of the median *χ* ^2^ drops steadily until around ∼250–300 orbits at which point diminishing returns begin to apply and the addition of more orbits does not significantly reduce the *χ* ^2^. Therefore, it could be said that, for the parameters selected, at least 200–300 uniformly distributed orbits should be considered before commenting conclusively on the measured cross‐tail distribution. It should be noted that the assumption of uniformly distributed orbits represents the simplest possible case, while in practice spacecraft trajectories often provide unevenly spread coverage. Figure 3b shows the median number of flux ropes observed as a function of the number of orbits, allowing the inference that the ∼250 orbit limit equates to a sample size of ∼60 flux ropes.

### 3.2 Varying System Parameters

**Figure 4:**
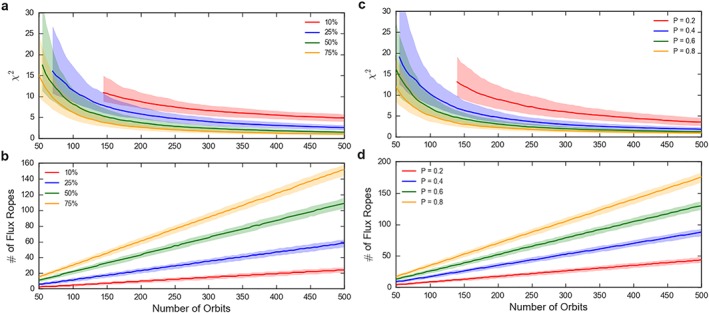
The median χ ^2^ of 1,000 iterations of the Monte Carlo (top) and the median number of flux ropes observed (bottom) for four different widths of neutral line (a and b) as a percentage of the width of the tail (4R_M_), and four different reconnection probabilities (c and d). The limits of the shaded regions represent the interquartile range. For the panels in which the width is varied (a and b) the probability is fixed at 0.5, while for the panels in which the probability is varied (c and d) the width is fixed at 50% of the tail width (i.e., 2R_M_). The remaining model parameters are as shown in Table 1.

The effects of varying several model parameters will now be explored. For example, one of the key model parameters is the width of the neutral line. Figures 2 and 3 were created with a uniform distribution of neutral line widths between 2 and 2.5*R*_*M*_ (Table 1). Figure 4a shows how the median *χ* ^2^ varies for a range of neutral line widths (with the probability of reconnection fixed at 0.5). It should be noted that the *χ* ^2^ metric cannot be evaluated if the true value for a bin is zero; therefore, the averages in Figures 4a and 4c only begin at the point at which every cross‐tail bin (in every iteration) had observed at least a single flux rope. For narrow neutral lines (e.g., those 10% of the model magnetotail: 0.4*R*_*M*_, in red) the *χ* ^2^ is both higher and drops slower than for the wider neutral lines. This is likely a result of the fact that smaller reconnection products will be observed less often by the spacecraft, and thus, the observed distribution is always less representative of the full distribution. This can be seen in Figure 4b, where the number of flux ropes observed for those spanning 10% of the tail only reaches ∼20 after 500 orbits. This is approximately the number that may be expected by simply taking the number of orbits and then multiplying through by the probability of reconnection and the fractional extent of the neutral lines (*N* ∼ 500 × 0.5 × 0.1 = 25). It should be noted that the effective sampling can be improved by increasing the width of the bins considered (i.e., the bin width could be said to be inappropriately narrow in Figure 2a).

Another interesting parameter to test is the probability of reconnection occurring during an orbital pass. Figure 4c shows how the median *χ* ^2^ varies for four selected probabilities (with the width fixed at 50% of the model tail width: 2*R*_*M*_). For a low probability (0.2, in red) the measured *χ* ^2^ is relatively high, once more linked to the low number of flux rope encounters (Figure 4d). In contrast, if the probability is high (e.g., 0.8, in yellow) then very few orbits are needed to adequately describe the tail, potentially as few as ∼150 orbits.

More generally, this technique allows the quantification of the common sense results: if the dynamic structures of interest are more azimuthally confined or less likely to be produced, then more orbits are required to constrain their distribution. Another interesting result that may be inferred from Figure 4 is that the *χ* ^2^ distributions do not correspond or scale linearly with the number of flux ropes observed; that is, there is not a predetermined number of flux ropes that is required to accurately assess the distribution (independent of the physical parameters of the structures). Additionally, orbits during which no dynamic product or evidence of reconnection is observed need to be accounted for when the spatial distributions are considered.

### 3.3 Orbit Selection

Sections 3.1 and 3.2 drew the spacecraft locations from uniform distributions (Table 1). However, uniform spacecraft coverage is often not possible for large surveys; therefore, the effects of uneven coverage will now be explored. In their recent survey of the Hermean tail Smith et al. (2017) used a catalog of 319 plasma sheet crossings (identified by ; Poh et al., 2017a).

**Figure 5:**
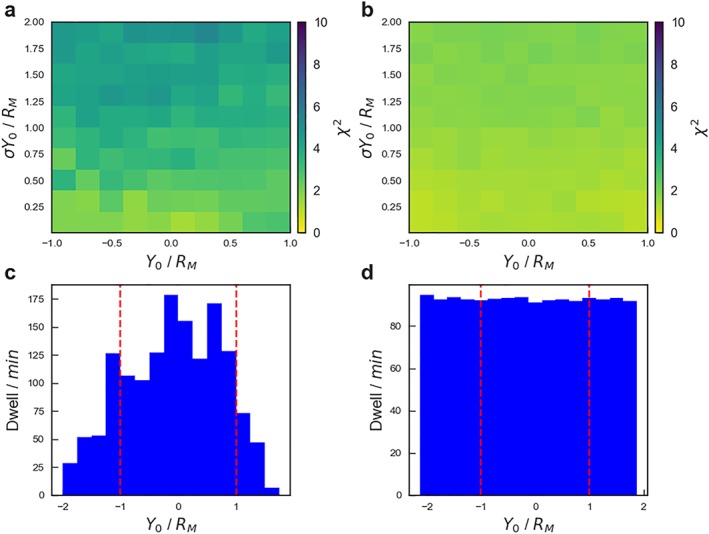
(top row) The mean χ ^2^ obtained between the intrinsic (true) and inferred spatial distributions after 319 orbits where the center of the neutral line is drawn from normal distributions described by Y_0_ and σ Y_0_. The means are calculated from a sample of 10,000 iterations. The results are shown for MErcury Surface, Space ENvironment, GEochemistry and Ranging's orbits as selected by (Poh et al., 2017a; a) and for randomly (and uniformly) distributed orbits (b). (bottom row) The cumulative dwell time within each spatial bin across the magnetotail for the orbits selected by (Poh et al., 2017a; c) and the mean dwell time per spatial bin for the uniformly distributed orbits (d). The red vertical dashed lines present in the lower panels represent the total width of the region plotted in the upper panels.

The effects of uneven spacecraft coverage will depend on the relative locations of both the spacecraft and the structures of interest. Therefore, for this investigation the uniform flux rope distributions are exchanged for normal distributions with a center and width defined by *Y*_0_ and *σ* *Y*_0_. The reconnection probability is set to 0.5, while the neutral line width remains between 2 and 2.5*R*_*M*_ (as above). Figure 5 compares the effectiveness of the orbit selection used by Smith et al. (2017; Figure 5a) with the same number of orbits (319) uniformly distributed over the magnetotail (Figure 5b). The quality with which the true distribution is recovered is quantified with a *χ* ^2^ metric (as above); this has been repeated 10,000 times for randomly selected combinations of *Y*_0_ and *σ* *Y*_0_. The results of the 10,000 iterations have then been averaged, and the mean per bin is presented in Figures 5a and 5b. The lower panels show the spatial sampling used by the Smith et al. (2017) survey (Figure 5c) and the mean of the uniformly distributed orbits (Figure 5d).

The 319 uniformly distributed orbits can be seen to well capture the underlying distribution (Figure 5b), with low (≤2) values of the *χ* ^2^ obtained for both narrow (low *σ* *Y*_0_) and wide distributions (high *σ* *Y*_0_) when the centers are located anywhere across the center of the magnetotail (−1*R*_*M*_≤*Y*_0_≤1*R*_*M*_). In contrast, the orbits used by Smith et al. (2017) can be seen to give poorer comparisons for most of the simulated distributions (Figure 5a). The reduced spacecraft coverage beyond *Y*_MSM_=±1*R*_*M*_ (Figure 5c) in particular results in more poorly recovered distributions at larger values of *σ* *Y*_0_ and toward *Y*_0_∼1*R*_*M*_.

However, even if the inferred distributions may not well represent the underlying distributions, it does not necessary follow that it is impossible to uniquely identify the intrinsic distribution. It is possible that use of the Monte Carlo method would still result in the inference of the correct underlying distribution. In the future, this technique could be used to evaluate the effectiveness of a given spacecrafts orbital coverage for observing statistical distributions of various transient features.

## 4 Spatial Distributions at Mercury

The model can be used to compare a given set of observations with various intrinsic distributions (each generated by unique set of system parameters). For this study the results of Smith et al. (2017) will be used for comparison. In order to make the comparisons valid either the model or the results of the survey require adjustment; for example, clusters of up to eight flux ropes were observed during a single plasma sheet crossing (a feature not present in the model). A mechanism could be added to the model to allow the generation of multiple flux ropes, however to keep the number of free parameters low (and minimize possible degeneracies) the results of Smith et al. (2017) have instead been reprocessed. This has been performed such that multiple detections within the same plasma sheet crossing are only counted as a single detection. For intervals when the orientation of flux ropes changed during a crossing, then the orientation is taken as that which dominated the interval.

First, the dawn‐dusk distribution of flux ropes will be considered. This will allow some of the physical parameters of the Mercury system to be estimated, for example, probability of reconnection and neutral line width. Once these parameters have been estimated, the model may be setup to provide an overall rate of flux rope detections that is consistent with observations. This will then allow the location of the NMNL to be explored by further investigation of the relative rates of planetward and tailward moving structures.

### 4.1 Dawn‐Dusk Distribution

First, the uniformly distributed spacecraft locations are replaced with those orbits performed by MESSENGER during the original survey (Smith et al., 2017). Second, the uniform distributions from which the neutral line locations were drawn (in sections 3.1 and 3.2) are exchanged for normal distributions. This allows parametrization in terms of a distribution center (*Y*_0_) and a distribution width (*σ* *Y*_0_), as in section 3.3. The final variables employed are the probability of reconnection during an orbital pass (*P*) and the width of the neutral lines (*W*_NL_). The model can then be run, for the MESSENGER orbits, for millions of iterations with random combinations of the four parameters (*Y*_0_, *σ* *Y*_0_, *P*, and *W*_NL_). Each iteration (consisting of the 319 orbits performed by MESSENGER) can be compared to the observed cross‐tail distribution from the survey (Smith et al., 2017), and a *χ* ^2^ metric derived for each combination of parameters. Three million parameter combinations were simulated, and the resulting parameter space smoothed with a histogram binning method. The number of simulations was observed to adequately sample the possible parameter space, while the smoothing removed stochastic variability between similar runs, allowing the underlying trends to be examined.

**Figure 6:**
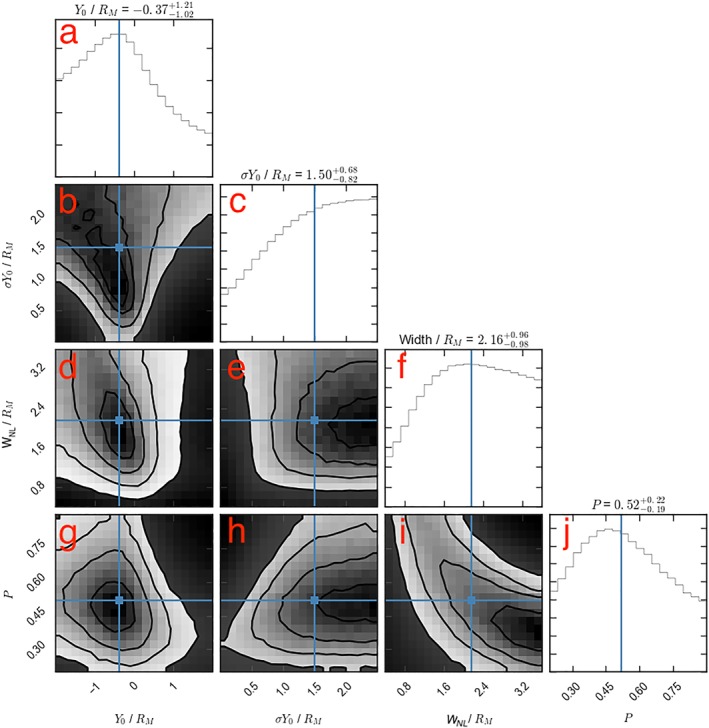
The posterior probability distributions of the model parameters: Y_0_, σ Y_0_, W_NL_, and P. The uppermost diagonal elements (a, c, f, and j) show the marginalized posterior probability distribution for each parameter in turn while the lower left panels (b, d, e, g, h, and i) show two‐dimensional projections for all combinations of parameters. The solid lines in the lower left show the one, two, and three sigma contours. The blue lines, points and values above the diagonal panels indicate the medians of each distribution. The confidence limits provided for the median values are 1σ.

The resulting four‐dimensional parameter space was then sampled using an affine‐invariant Markov chain Monte Carlo (MCMC) ensemble sampler (Foreman‐Mackey et al., 2012), in order to estimate the Bayesian posterior probability density functions (PPDFs): the probability distribution of the variables given the evidence presented by the sampling. Figure 6 shows the results of the MCMC sampling. The six panels in the lower left (b, d, e, and g–i) represent the one, two, and three sigma contours projected onto all possible combinations of two parameters. The panels along the uppermost diagonal (a, c, f, and j) represent the PPDF functions marginalized for each of the four parameters considered. The blue dots/lines represent the medians of the marginalized PPDFs. It should be noted that the medians may not be colocated with visible peaks if the full distributions are not present within the simulation limits; therefore, it is perhaps more constructive to draw conclusions from the peaks and shapes of the marginalized distributions (if they extend beyond the simulated parameter space).

First, the distribution in Figure 6a shows that the results of the survey are most consistent with neutral line distribution marginally offset dawnward of midnight ( $Y_{0}=-0.37_{-1.02}^{+1.21}\;R_{M}$), though the midnight meridian is within 1*σ*. The results are also most consistent with a relatively broad neutral line distribution (Figure 6c), indicating a substantial amount of variability between orbital passes. The sampling provided by the selected MESSENGER orbits (Figure 5c) has been shown to poorly recover broad distributions: this likely results in the lack of an “edge” to the distributions on the broad side (with large *σ* *Y*_0_).

Second, the median width of the neutral line is found to be $2.16_{-0.98}^{+0.96}\;R_{M}$, just over half the width of the model magnetotail (Figure 6f). However, this should be regarded as an upper limit as there is no consideration of the magnetopause boundary, and so the effective width of the neutral line could be up to a factor of 2 smaller (depending on the location of the neutral line center). It is also clear from the shape of the *W*_NL_ distribution in Figure 6f that larger neutral lines (i.e., to the right of the peak of the distribution) are more consistent with the survey results, rather than those $≲1.6\;R_{M}$. Finally, from the marginalized distributions, the median probability of a neutral line forming during a plasma sheet crossing is found to be $0.52_{-0.19}^{+0.22}$. This result is intuitive: Smith et al. (2017) found that during 39% of crossings flux ropes were observed. Accounting for occasions where the spacecraft was not colocated with the neutral line will result in a fraction greater than 39%.

Figure 6 also shows the covariances between the parameters. For example, from Figure 6d, if the width of the neutral line is larger, then the distribution center (*Y*_0_) is required to be offset further toward dawn. This is shown by the diagonal slope formed by the probability contours, from upper left to middle bottom. This is necessary to explain the relative lack of observations duskward of ∼1*R*_*M*_ (Smith et al., 2017). If the neutral lines are wider, then a more central distribution would result in the observation of significant numbers of flux ropes close to dusk. The same relationship can be seen in the *σ* *Y*_0_ versus *Y*_0_ panel (Figure 6b), where the contours slope from upper left to lower middle. Physically, this can be interpreted as a broader distribution requiring that the center be offset further toward dawn. Finally, a classically expected degeneracy is quantified by the panel showing the projection onto width (*W*_NL_) versus probability (*P*) space (Figure 6i): If there is a greater probability of reconnection occurring, then the neutral lines are required to be narrower and vice versa.

### 4.2 Downtail Neutral Line Location

The previous section allowed the basic parameters of the model to be estimated, that is, those which provide a rate of flux rope observations that best match the survey results. The downtail location of the neutral line can now be investigated by using the derived parameters and comparing the relative rates of the tailward and planetward moving distributions. For this, the neutral line location is parameterized in terms of a distribution center (*X*_0_) and a width (*σ* *X*_0_; which physically corresponds to variation between individual orbits).

**Figure 7:**
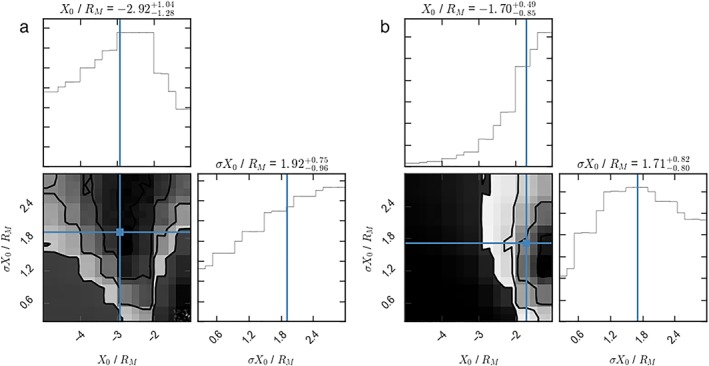
The posterior probability distributions for the tailward (a) and planetward (b) distributions of flux ropes. The formats are the same as for Figure 6.

Over a million simulations were performed with random selected combinations of *X*_0_ and *σ* *X*_0_, sufficiently sampling the parameter space. The planetward and tailward distributions were each compared to the respective results from the survey of Smith et al. (2017), and two *χ* ^2^ metrics evaluated (for the planetward and tailward distributions separately). As with section 4.1, the results were smoothed using a histogram and the parameter space sampled using an affine‐invariant MCMC sampler (Foreman‐Mackey et al., 2012). The results are displayed in Figures 7a and 7b for the tailward and planetward moving distributions, respectively. The formats are the same as for Figure 6.

The results for the tailward distribution (Figure 7a) give a median neutral line location of $X_{0}=-2.92_{-1.28}^{+1.04}$ and favor a relatively broad distribution (in *σ* *X*_0_). This result is consistent with a previous statistical study: Poh et al. (2017a) inferred the location to be on average at ∼−3*R*_*M*_ (using an independent method).

However, the results for the comparison of the planetward moving distribution (Figure 7b) are not consistent with that found for the tailward population, with a median neutral line location of $-1.70_{-0.85}^{+0.49}$ appearing most consistent. The X‐line location inferred from the tailward moving population (*X*_0_∼−3*R*_*M*_) would result in too high a rate of planetward moving flux ropes, much greater than is observed. Therefore, the X‐line is inferred to be closer to the planet. It is also clear that simply increasing the variability in the location of the X‐line (i.e., increasing *σ* *X*_0_, moving up in Figure 7b) is insufficient to account for this effect. In other words, the contours in Figure 7b do not allow the X‐line to move deeper into the tail (left) if the variability in location is greater (*σ* *X*_0_ increases). The lack of self‐consistency in the neutral line location suggests that there is some physics of the underlying system not captured by the simple parameterization.

**Figure 8:**
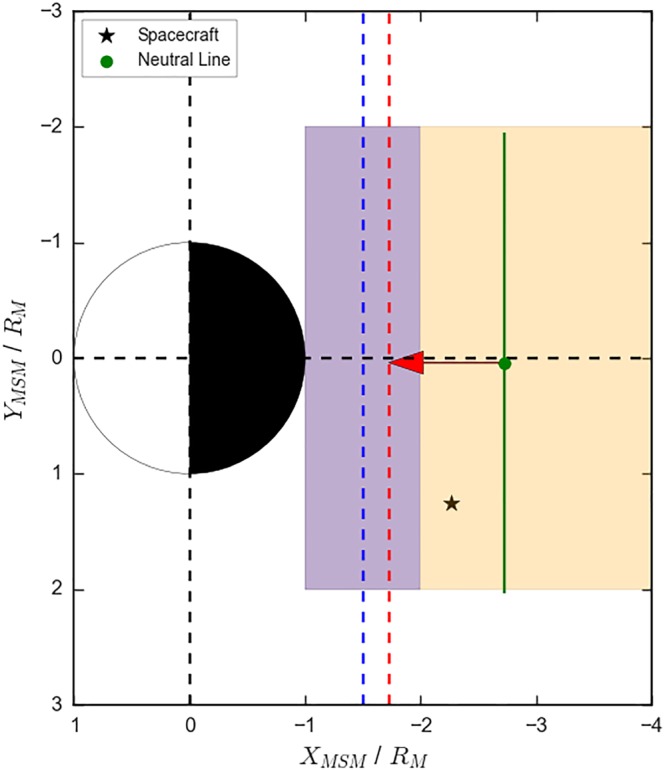
Schematic describing the two‐dimensional model setup, adapted from that in Figure 1. The additions are shown by a maximum travel distance, indicated with the red arrow and vertical dashed line, and a distance of closest approach indicated with a blue shaded region and vertical dashed line.

To investigate this, additional parameters are added to the model. The first consideration is that there is perhaps some maximum distance that the flux rope can travel from the X‐line, at which point it becomes unrecognizable as a flux rope, parameterized as a distance *A*. Physically, this could correspond to the flux rope becoming distorted, such that it is not well approximated by the force free model, or perhaps forming a dipolarization front (e.g., Lu et al., 2015). This travel distance is represented by the red arrow and dashed line in Figure 8. Therefore, in order to observe the flux rope, the spacecraft would have to be located tailward of the red dashed line. The second mechanism added to the model is a distance of closest approach to the planet by the flux rope, parameterized with *X*_Min_, and some variation in this value (*σ* *X*_Min_). Physically, this could represent the distance at which the flux rope halts its planetward motion, re‐reconnecting with the planetary field (Slavin et al., 2003). This region is represented by the blue dashed line (*X*_Min_) and shaded region (*σ* *X*_Min_) in Figure 8. As with the maximum travel distance (*A*), the spacecraft must be located tailward of the distance of closest approach in order to observe a flux rope.

**Figure 9:**
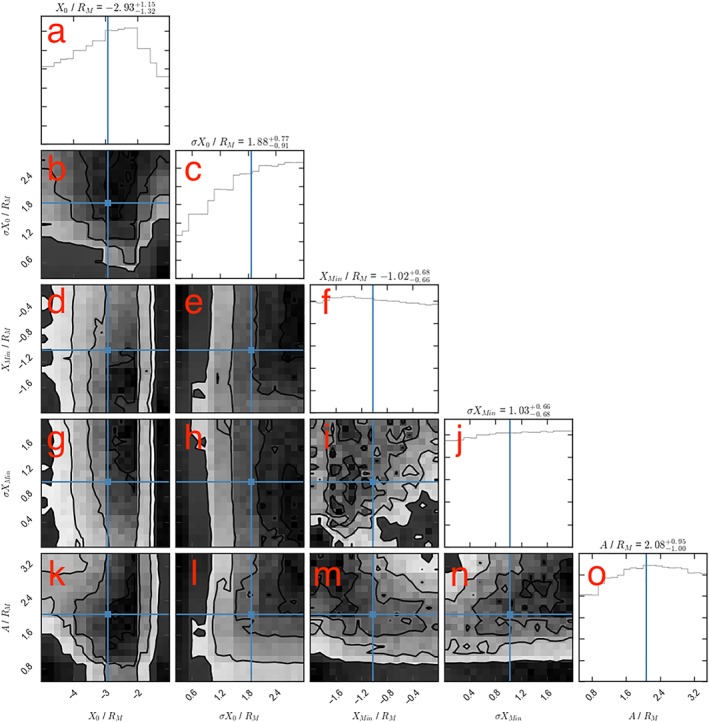
The posterior probability distributions of the model (X_0_ and σ X_0_) including parameters for potential loss mechanisms planetward of the X‐line (A, X_Min_, and σ X_Min_). In similar format to Figures 6 and 7, the uppermost diagonal panels (a, c, f, j, and o) show the marginalized posterior probability distributions for each parameter, while the lower left panels (b, d, e, g, h, i, k, l, m, and n) show the two‐dimensional projections for all parameter combinations.

Figure 9 shows the results of the model with the addition of these parameters (for the planetward distribution). The addition of the loss terms has reduced the median value of *X*_0_ such that it is now fully consistent with both the tailward distributions in Figure 7a and previous studies (e.g., Poh et al., 2017a; with a median $X_{0}=-2.93_{-1.32}^{+1.15}$). This suggests that some form of dissipation planetward of the neutral line is fundamentally important at Mercury within the region surveyed by MESSENGER.

Once more, the median values quoted above the diagonal panels in Figure 9 should be regarded with a degree of caution as the full distributions are not within the simulated parameter space. It is also clear that the parameterization of the loss terms is not entirely physical: the marginalized distributions do not show a clear peak for *X*_Min_, *σ* *X*_Min_, or *A*. However, the addition of these dissipation mechanisms does allow the X‐line location to be self‐consistent. Additionally, a faint relationship is observed between *X*_Min_ and *A* (Figure 9m): increasing the size of the quasi‐dipolar region (decreasing *X*_Min_) increases the maximum travel distance (*A*) that is consistent with the observations. Physically this would correspond to a larger “quasi‐dipolar region” negating the requirement for a maximum travel distance, and vice versa.

## 5 Discussion

A Monte Carlo model has been presented which allows the orbital sampling of a single spacecraft to be investigated. The model was tailored to investigate Mercury's magnetotail and used to evaluate a recent survey of MESSENGER spacecraft data. The model presented has confirmed that, accounting for the orbital sampling of MESSENGER and the finite width of magnetic flux ropes, the effects of a slight dawn‐dusk asymmetry in the location of the Mercury's magnetotail neutral line are present in the observations of a recent flux rope survey (Smith et al., 2017). The inferred neutral line asymmetry (e.g., Sun et al., 2016) has previously been linked to asymmetries in the plasma population (Poh et al., 2017b). Mercury's plasma sheet has been found to predominantly consist of H^+^ and Na^+^, with the Na^+^ density determined to peak premidnight (Delcourt, 2013; Gershman et al., 2014; Raines et al., 2013). The presence of such heavy ions (e.g., Na^+^) has been suggested to increase the growth rate of the tearing mode instability (Baker et al., 1982), thereby causing reconnection. Conversely, it has also been suggested that the presence of the heavier ions will reduce the mean Alfvén speed, reconnection inflow velocity, and therefore the rate of reconnection (Shay & Swisdak, 2004). The results of this investigation and previous studies (e.g., Baker et al., 2016; Dewey et al., 2017; Lindsay et al., 2015; Poh et al., 2017b; Smith et al., 2017; Sun et al., 2016) suggest that the latter mechanism may dominate in the Hermean tail.

In order to reproduce the observed planetward and tailward moving distributions, dissipation terms were required planetward of the neutral line. These terms could be physically explained as mechanisms that would re‐reconnect the flux rope with the planetary field (Slavin et al., 2003) or distort the structure of the flux rope in such as way that it is not recognizable (e.g., forming a dipolarization front; Lu et al., 2015).

## 6 Conclusions

A Monte Carlo‐based analysis technique has been presented and applied to a single‐spacecraft survey of Mercury's magnetotail. First, synthetic, randomly distributed orbits were tested to determine the number of orbits required to obtain a good estimate of the underlying intrinsic distributions of magnetotail flux ropes. The required number of orbits was shown to be heavily dependent upon the properties of the system and the flux ropes themselves, for example, the width of the structures and the probability of their occurrence. The efficacy of two different orbital sampling regimes were compared; uniformly distributed orbits were found to best infer the majority of intrinsic distributions tested.

Second, many iterations with different combinations of model parameters were performed and compared to the results of a recent survey (Smith et al., 2017). The survey results were found to be most consistent with an neutral line that is offset dawnward of midnight by $-0.37_{-1.02}^{+1.21}\;R_{M}$. Azimuthally wider flux ropes (e.g., ≥2*R*_*M*_) were found to be more consistent with the results, rather than narrower structures. The statistical downtail location of the neutral line was then probed. The distribution of tailward moving flux ropes allowed the recovery of a statistical location consistent with previous studies (e.g., Poh et al., 2017a). However, the distribution of planetward moving structures returned a result that was both inconsistent with previous work in the literature and with the results obtained from the comparison to the tailward moving distribution. This discrepancy could be resolved with the addition of parameters describing dissipation mechanisms planetward of the reconnection site (e.g., a “maximum travel distance” or “distance of closest approach”).

This work allows the effects of orbital sampling from a single spacecraft to be explored, suggesting the required orbital coverage (given properties of the system). It also allows the inference of the global properties of the system that are most consistent with a set of observations. This type of analysis, with specific adaptation, could be useful for both future statistical studies at Mercury and at other planets as well as for mission/trajectory design.
